# Tick-Borne Diseases and Pregnancy: A Narrative Review Evaluating Pregnancy Complications Caused by Tick-Borne Diseases

**DOI:** 10.3390/tropicalmed9110254

**Published:** 2024-10-24

**Authors:** Michael W. Curtis, Job E. Lopez

**Affiliations:** 1 Department of Pediatrics, National School of Tropical Medicine, Baylor College of Medicine, Houston, TX 77030, USA; job.lopez@bcm.edu; 2 Department of Molecular Virology and Microbiology, Baylor College of Medicine, Houston, TX 77030, USA

**Keywords:** tick-borne disease, pregnancy, Lyme disease, tick-borne relapsing fever, anaplasmosis, Rocky Mountain spotted fever, tick-borne encephalitis, Crimean-Congo hemorrhagic fever, babesiosis

## Abstract

Ticks are vectors of public health concern because the pathogens they transmit can cause detrimental diseases in humans. Lyme disease, tick-borne relapsing fever, human granulocytic anaplasmosis, Rocky Mountain spotted fever, tick-borne encephalitis, Crimean-Congo hemorrhagic fever, and babesiosis are some of the most common diseases caused by the pathogens transmitted by ticks. The overlap between human activities and tick habitats is growing, contributing to an increase in tick-borne disease cases. Unfortunately, pregnancy as a risk factor for tick-borne diseases is largely ignored. In this narrative review we use case reports, epidemiological studies, and animal studies to evaluate the maternal, pregnancy, and fetal outcomes caused by Lyme disease, tick-borne relapsing fever, human granulocytic anaplasmosis, Rocky Mountain spotted fever, tick-borne encephalitis, Crimean-Congo hemorrhagic fever, and babesiosis during pregnancy.

## 1. Introduction

Ticks are hematophagous ectoparasites that continue to be a public health concern due to the pathogens they transmit. Ticks are monophyletic and divided into the *Ixodidae*, *Argasidae*, and *Nuttalliellidae* families. *Nuttallielidae* is composed of a single species, *Nuttallielidae namaqua*, which has only been found in Africa [1]. Both *Ixodidae* and *Argasidae* are globally distributed and composed of hundreds of tick species [2,3]. While it is unknown if *N. namaqua* harbors or transmits human pathogens [1], tick species from both *Ixodidae* and *Aragasidae* have been known to transmit human pathogens since the early 1900s [4,5]. Lyme disease, anaplasmosis, spotted fever rickettsioses, ehrlichiosis, babesiosis, tick-borne encephalitis, Crimean-Congo hemorrhagic fever, *Borrelia miyamotoi* disease, and Powassan virus disease are examples of the diseases caused by the pathogens transmitted by *Ixodidae* ticks. Several viruses that cause disease in humans and animals are transmitted by *Argasidae*, but relapsing fever-causing spirochetes are the most known pathogens vectored by these ticks. 

People continue to be at risk of tick-borne disease due to the growing overlap between human and tick populations. Forestry and outdoor occupations in tick endemic areas place individuals at an increased risk of exposure to infected vectors [6]. Additionally, robust tick populations have been observed in urban green spaces all around the world. Urban green spaces are natural or vegetated areas within or close to urban settings [7], and are vital parts of urban sustainability and associated with health benefits [8]. Unfortunately, studies have reported the presence of pathogens in the ticks and wildlife in urban green spaces globally [9,10,11,12,13,14,15,16]. Concerningly, over half of the world’s population lives in urban settings with projections estimating that number to grow to ~70% by 2050 [17,18], suggesting that tick-borne disease risk will continue to rise. We have also observed soft ticks inhabiting peridomestic settings in underdeveloped regions [16,19,20]. The increased overlap of ticks and tick-borne pathogens with human populations warrants enhanced investigations into surveillance and pathogenesis, especially for the vulnerable subpopulations that are at an increased risk of severe adverse outcomes [21,22,23]. 

While work has progressed in understanding the disease manifestations and pathogenesis of tick-borne pathogens, pregnancy as a risk factor has largely been ignored. Altered immune system physiology during pregnancy may lead to an increased susceptibility or severity of infectious diseases [24,25,26]. For example, pregnant individuals and fetuses are at an increased risk for severe outcomes from influenza, measles, malaria, and other pathogens [24,27,28,29,30,31,32,33]. Investigating pregnancy as a risk factor for tick-borne diseases is warranted because the overlap between human populations and tick habitats will likely lead to an increased risk of disease among pregnant individuals. Additionally, the occupational hazards of pregnant workers may increase the risk of exposure [6]. However, our knowledge regarding the disease manifestations and outcomes for tick-borne disease during pregnancy is very limited. The aim for our narrative review is to summarize the available data pertaining to Lyme disease, tick-borne relapsing fever, anaplasmosis, Rocky Mountain spotted fever, Crimean-Congo hemorrhagic fever, tick-borne encephalitis, and babesiosis during pregnancy. The information presented is from case reports, animal models, and epidemiological studies (Table 1).

## 2. Lyme Disease

Lyme disease is the most common tick-borne disease in the United States and Europe and is caused by species of spirochetal bacteria known collectively as *Borreliella* (*Borrelia*) *burgdorferi sensu lato*. In the United States, *B. burgdorferi* is the primary cause of Lyme disease, while *Borrelia afzelii* and *Borrelia garinii* are the species in Europe [50]. Lyme disease-causing spirochetes are transmitted by species of *Ixodes* ticks and cause multisystem symptoms such as fever, fatigue, headache, and chills during early infection [51,52,53,54,55]. Additionally, about 60–90% of patients develop a rash emanating from the site of the tick bite known as erythema migrans [51,52,53,55]. If an early infection is not treated, the spirochetes disseminate and cause arthritis in the large weight-bearing joints, neurologic complications, and carditis [51,56,57]. Case reports, prospective and retrospective studies, animal studies, and a systemic review have provided critical insights into the maternal and perinatal disease outcomes following Lyme disease during pregnancy. 

Studies investigating gestational Lyme disease have centered on two main topics: (1) maternal and perinatal adverse outcomes and (2) the vertical transmission of Lyme disease-causing spirochetes from mother to offspring. Case reports provided the first insights into the pregnancy complications associated with Lyme disease through the description of the maternal and perinatal outcomes. In the early 1980s, the Centers for Disease Control (CDC) reported 19 cases of gestational Lyme disease [58]. Perinatal adverse outcomes (defined by the authors as intrauterine fetal demise in the second trimester, prematurity, and developmental delay with cortical blindness) were found in five of the cases, while the other fourteen cases resulted in healthy births [58]. Additional case reports and studies have described erythema migrans, arthralgia, arthritis, borrelial lymphocytoma, and neurologic manifestations as the symptoms of maternal infection, while the fetal outcomes ranged from normal healthy babies to mild (angiomatous patches) and severe outcomes (fetal death, pre-term birth, stillbirth, and perinatal death) [59,60,61,62,63,64,65,66,67,68,69,70,71] and are reviewed in [72]. 

The first evidence of human maternal-to-fetal transmission of *B. burgdorferi* was described in a case report from Wisconsin in 1983 [66]. The mother was diagnosed with Lyme disease five days postpartum due to the development of arthritis in her wrist and a serum IgG antibody titer to *B. burgdorferi* of 1:128 [66]. Her baby, born at 35 weeks, died within two days due to widespread congenital cardiac abnormalities. An autopsy revealed spirochetes in his spleen, renal tubules, and bone marrow [66]. It is important to note that while this case was attributed to *B. burgdorferi*, no isolation or sequence data were collected to verify the speciation. Additionally, at the time of this case, *Borrelia mayonii*, *Borrelia miyamotoi*, and *Borrelia johnsonii* had not been identified yet. *B. mayonii* is a Lyme disease-causing spirochete endemic to the Upper Midwest, while *B. miyamotoi* and *B. johnsonii* are relapsing fever-causing spirochetes that have been found in Wisconsin [73,74]. Moreover, anti-relapsing fever *Borrelia* antibodies have been observed to cross-react with *B. burgdorferi* antigens [75,76,77]. The vertical transmission of Lyme disease-causing spirochetes was described in additional cases through the staining of spirochetes with the anti-*B. burgdorferi* monoclonal antibody H5332, silver stain, culturing, or PCR [64,67,69,72,78]. These case reports spurred further investigations into the potential of *B. burgdorferi* to cause adverse outcomes following infection during gestation.

Large epidemiology studies have aimed to address the disease progression and outcomes of Lyme disease-causing spirochete infection during pregnancy. Between the 1980s and 1990s, prospective, retrospective, and sero-survey studies provided needed insights into the perinatal outcomes of gestational Lyme disease. A prospective study enrolled just under 2000 pregnant women from Westchester County, NY. The enrolled women completed a survey, and blood was collected at their first prenatal visit and at delivery to determine the presence of anti-*B. burgdorferi* antibodies [79]. Based on the survey and serum testing, the women were grouped according to the time of the Lyme disease diagnosis in relation to the pregnancy (during (*n* = 15), <1 year prior (*n* = 29), >1 year prior (*n* = 21), and unknown timing (*n* = 14)) and the never-diagnosed controls (*n* = 1818) [79]. No differences in fetal deaths, birth weights, lengths of gestation, or congenital malformations were found between any of the groups reporting a Lyme disease diagnosis or the controls [79]. 

A retrospective study investigated if there was an association between Lyme disease and congenital heart disease. The authors reported that the incidence of maternal Lyme disease during or before pregnancy did not differ between cohorts of children diagnosed with a congenital heart defect and children without a heart defect from the same pediatric cardiology service in New York [80]. This suggested that Lyme disease was not associated with congenital heart defects. 

A sero-survey of over 5000 samples of cord blood from infants born to mothers within an endemic (Westchester County, New York, *n* = 2504) or nonendemic (Rockland County, NY, *n* = 2507) region of Lyme disease determined whether Lyme disease caused congenital malformations [81]. The cord blood from the endemic region was 10× more likely to test positive for anti-*Borrelia* antibodies via ELISA than the cord blood from the nonendemic region [81]. There were significantly more cardiac abnormalities in the endemic region compared to the nonendemic; however, there was no difference in abnormalities or mean birth weights between the sero-positive and sero-negative cord blood cohorts within the endemic cohort [81]. Therefore, it was difficult to conclude whether Lyme disease causes congenital abnormalities from this study. 

A recent international cross-sectional survey investigated the impact Lyme disease has on pregnant individuals and their children [82]. The authors surveyed 691 eligible participants recruited from the websites and social media platforms of the McMaster Midwifery Research Centre and Lyme disease-focused organizations [82]. The pregnancies (*n* = 1154) from the surveyed participants were separated into the following groups: “Probable Treated LD (*n* = 135)”, “Probable Untreated LD (*n* = 124)”, “Possible Untreated LD (*n* = 480)”, “No Evidence of LD *n* = 364)”, and “Unclear (*n* = 51)” [82]. The authors found the “No Evidence of Lyme disease” group reported significantly fewer complications compared to the Probable Treated, Probable Untreated, and Possible Untreated Lyme disease groups (*p* < 0.01) [82]. Rash, hypotonia, and respiratory distress were reported in newborns within the first two weeks after birth more frequently in the Probable Untreated group compared to the Probable Treated, Possible Untreated, and No Evidence of Lyme disease groups (*p* < 0.01) [82]. While this report contributes to the growing knowledge surrounding the consequences of Lyme disease during pregnancy, the authors acknowledged some limitations. For example, some participants recalled events from 10–70 years prior to the survey; therefore, discrepancies may have arisen [82]. Also, based on the recruitment from Lyme disease-focused organizations, the participants were likely to report chronic/late-stage disease more often than a random group of Lyme disease patients [82]. 

A systematic review of the 29 case reports or series and 17 epidemiology studies published globally investigated the association between Lyme disease and adverse maternal or perinatal outcomes [83]. Despite adverse outcomes being reported in 59 cases, the authors did not find any difference in the prevalence of adverse pregnancy outcomes between the populations exposed to Lyme disease (defined by the individual authors as gestational LD, history of LD, tick bites, or residence in an endemic area) and the control populations [83]. When the authors performed a meta-analysis of nine studies that investigated the pregnancy outcomes of treated vs. untreated gestational Lyme disease, they found indirect evidence that Lyme disease-causing spirochete infection may cause adverse pregnancy outcomes. The meta-analysis revealed fewer adverse outcomes in the treated pregnant populations compared to the untreated pregnant populations [83]. Taking together the discrepancy between the case reports and epidemiologic studies, the added complexity of the cross-sectional survey, and the indirect evidence of increased adverse outcomes of untreated compared to treated gestational Lyme disease, a causal link between infection with Lyme disease spirochetes during pregnancy and adverse pregnancy outcomes remains controversial. Future large-scale prospective studies are warranted. 

Two animal studies investigated the risk of adverse perinatal outcomes following gestational Lyme disease. One study investigated the perinatal outcomes following acute (mother infected on gestation day 4) and persistent (infection 3 weeks prior to mating) infection of *B. burgdorferi* in C3H/HeN mice [34]. *B. burgdorferi* was administered via intradermal inoculation at a dose range of 2 × 10^5^ to 2 × 10^7^ cells. In total, 12% of the 280 gestational sacs resulted in fetal death across all doses of the acutely infected mothers. No fetal deaths were observed in the persistently infected and control groups [34]. No differences between the doses were observed [34]. 

A second study investigated the perinatal outcomes following repeated intradermal inoculation of *B. burgdorferi* in the footpad of pregnant canines. The inoculations began on day 1 of proestrus and were repeated every two weeks until delivery. No differences in the litter size or pup weight were observed between the infected and uninfected cohorts. The authors did note that 3 of 9 infected female canines contained more implantation sites than pups delivered, while this was not observed in the noninfected control canines, suggesting a possibility of fetal absorption [35]. Together, these animal studies suggest gestational infection with *B. burgdorferi* may cause adverse pregnancy outcomes.

Additional animal studies disagreed regarding the occurrence of the vertical transmission of *B. burgdorferi*. An early study infected five pregnant Lewis rats with *B. burgdorferi* at a dose of 1 × 10^6^ on day 4 of gestation [36]. Despite all the maternal rats either seroconverting or having positive splenic cultures on day 20 of gestation, no vertical transmission of *B. burgdorferi* was observed [36]. Another study investigated vertical transmission by monitoring field-caught pregnant white-footed mice (*Peromyscus leucopus*) through delivery [38]. Upon delivery, naïve ticks were placed on the individual mothers and offspring for xenodiagnoses. The ticks from all of the mothers became infected following engorgement, but none of the ticks that fed on any of the offspring became infected, suggesting that *P. leucopus* does not vertically transmit *B. burgdorferi* from mother to offspring [38]. The authors noted a few offspring did have anti-*Borrelia* antibodies, demonstrating the possibility of the maternal transfer of anti-*Borrelia* antibodies [38]. An additional study performed on Syrian hamsters failed to detect vertical transmission of *B. burgdorferi* from mother to offspring [37]. Conversely, several animal studies reported the vertical transmission of *B. burgdorferi*. One study found that although no *B. burgdorferi* DNA was detected from 10 fetuses (euthanized on gestation day 14–16) from the mice infected with *B. burgdorferi* on gestation day 4, *B. burgdorferi* DNA was amplified in one of three fetuses and two of three placentas from the mice infected with *B. burgdorferi* five days prior to mating and euthanized on gestation day 14 [34]. Fetuses from infected, field-caught *Mus musculus* and *Peromyscus domesticus* on a farm in Wisconsin were assessed for *B. burgdorferi* infection via culturing [39]. Two fetuses from an infected pregnant *M. musculus* and one fetus from an infected pregnant *P. domesticus* were found to be infected with *B. burgdorferi* [39]. A third study reported the vertical transmission of *B. burgdorferi* in canines following the repeated (beginning on day 1 of proestrus and repeated every two weeks until delivery) intradermal inoculation of 1000 spirochetes [35]. The pups from eight of ten litters from the infected mothers were either PCR-positive or culture-positive for *B. burgdorferi*, indicating that upon repeated inoculations, *B. burgdorferi* is vertically transmitted from mother to offspring in canines [35]. Based on all of these animal studies, the vertical transmission of *B. burgdorferi* remains controversial and undefined. 

## 3. Tick-Borne Relapsing Fever

Tick-borne relapsing fever (TBRF) is a globally distributed disease caused by species of spirochete bacteria in the *Borrelia* genus [84,85]. Most TBRF-causing spirochetes are transmitted by *Aragasidae* ticks, while one species is transmitted by *I*. *scapularis*. In the mammal, TBRF-causing spirochetes cyclically reach high densities in the blood, commonly known as spirochetemia. This is due to the antigenic variation of the variable major protein (VMP) on their outer membrane [86,87,88]. The mechanism of antigenic variation has been reviewed previously [89,90,91]. Briefly, there is expression site for the gene encoding VMPs (*vmp*), and multiple silent *vmp* alleles that can be integrated into the expression site [92,93]. The genomes of TBRF *Borrelia* known to cause disease in North America contain between 94 and 154 silent *vmp* alleles [94]. As the spirochetes begin to replicate in the host blood, one VMP-variant becomes predominant. The host mounts an antibody response against the predominant VMP-variant, clearing that population. However, a silent *vmp* allele recombines into the expression site and a new variant emerges in the blood, inducing a new antibody response and subsequent clearance by the host. This dynamic between VMP-variant emergence and antibody response can continue for months if untreated [89]. During spirochetemic episodes, the TBRF infection causes repeated high fever, illness, headache, chills, myalgia, neurological complications, pregnancy complications, and potential death, if the infection goes untreated [95,96]. 

Despite reports of ticks infected with TBRF-causing spirochetes being found on all continents except for Australia and Antarctica, most of the epidemiological studies have been conducted in Africa, while TBRF is rarely considered in North and South America, Europe, and Asia [97,98,99]. Studies in Africa have shown the TBRF incidence rate in endemic areas is among the highest for bacterial infections on the continent [100]. Case reports and studies from Africa describe abortion, pre-term delivery, and perinatal death as severe outcomes of TBRF *Borrelia* infection during pregnancy [101,102,103,104,105,106]. A case-controlled study performed in Tanzania from 1985–1995 investigated the maternal and perinatal outcomes caused by gestational TBRF [101]. The investigators compared 137 TBRF cases in pregnant females to 120 TBRF cases in non-pregnant females [101]. This study revealed the rate of giving birth during a spirochetemic episode was 58% and the perinatal mortality rate was 43.6% [101]. The authors found that perinatal death positively correlated with low birth weight and low gestational age at birth [101]. They also found that the spirochetes were able to replicate to higher densities within infected, pregnant females compared to infected, non-pregnant females. While the level of spirochetemia positively correlated with the risk of giving birth during a spirochetemic episode, it did not correlate with perinatal mortality [101]. 

Another study from Tanzania analyzed 27 cases of TBRF during pregnancy to identify risk factors associated with severe disease outcomes [107]. This study observed the level of spirochetemia correlates with severe disease outcomes such as miscarriage, fetal death, pneumonia, hepatitis, or severe jaundice with tender hepatomegaly and neonatal asphyxia [107]. The authors did not find any correlation between the age of the pregnant female and the disease outcome, but observed that infections during early gestation (<7 months) and late gestation (>7 months) resulted in more severe outcomes than infections around 7 months of gestation [107]. However, due to the low case numbers in each gestation stage (<10), more studies are needed to determine if this trend is biologically relevant. Case studies from North America and the Middle East report similar disease outcomes following TBRF infection during pregnancy [103,108,109,110,111,112,113], suggesting a common disease pathology among TBRF spirochetes found throughout the world. The maternal-to-fetal transmission of TBRF spirochetes has been observed [103,104,108,109,114], but the pathogenesis of perinatal mortality is unclear because few studies have investigated the mechanisms. 

The underlying cause of the adverse perinatal outcomes associated with TBRF infection during pregnancy is unknown. Only a single animal study has investigated the impact of TBRF *Borrelia* infection during pregnancy [40]. The authors assessed the impact *Borrelia duttonii* (an African TBRF *Borrelia* species) infection has on maternal and perinatal health following the subcutaneous injection of pregnant mice on day nine of gestation [40]. On day 18 of gestation, the authors euthanized the mice and characterized the fetuses. They observed that the fetuses from infected mothers weighed 18% less and had increased rates of abnormalities (defined by the authors as ashen skin, blotchy complexion, the absence of visible blood in the vessels, and/or the presence of apparent internal hemorrhages) than the fetuses from uninfected mothers [40]. *B. duttonii* was detected via immunofluorescence within the labyrinth area of the placenta and within the fetuses, demonstrating the transplacental transmission of TBRF spirochetes from mother to fetus for the first time [40]. The authors also found that fetal weight inversely correlated with the mother’s spirochetemia level [40]. However, unlike what is observed during human TBRF infection during pregnancy, the female mice had lower spirochetemia levels compared to the non-pregnant female mice, and there was no evidence of increased fetal death among the infected pregnant mice. 

## 4. Human Granulocytic Anaplasmosis

Human granulocytic anaplasmosis (HGA) is an emerging tick-borne disease found in the United States, Asia, and Europe that is caused by *Anaplasma phagocytophilum*, an obligate intracellular bacterium [115,116]. *A. phagocytophilum* is transmitted by tick species from the *Ixodes* genus and causes symptoms ranging from mild, nonspecific fever illness to severe human disease [115]. A systematic review of published cases determined the case fatality rate among immunocompromised individuals (18.2%) is higher than among immunocompetent individuals (4.2%) [117]. The increased severity among immunocompromised individuals highlights the interest in investigating the HGA outcomes among pregnant individuals. 

While *A. phagocytophilum* has been implicated in abortion and stillbirths in ruminants infected during pregnancy [118,119,120], its disease pathology in humans is poorly understood. Less than 15 cases of *A. phagocytophilum* infection during pregnancy have been reported [121,122,123,124,125]. The symptoms in the pregnant infected individuals were similar to those reported in non-pregnant individuals: fever (38.4–40.6 °C), headache, and myalgia and/or arthralgia [121,122,123,124,125]. Most reported cases of HGA were diagnosed between 1997 and 2006 at the Westchester Medical Center [122]. The patients ranged in gestational age at diagnosis from 10 weeks to 34 weeks, and the symptoms included fever, headache, myalgia, and/or arthralgia. The symptoms resolved in all patients either with or without the need for antibiotic treatment [122]. Patient follow-ups revealed normal health and development among all children [122]. While most published cases of *A. phagocytophilum* infection during pregnancy presumably occurred by tick bite, there has been one report of HGA transmission to a pregnant individual via blood transfusion [126]. A 34-year-old pregnant female with a β-thalassemia trait received nine units of irradiated, leukoreduced red blood cells over a course of 4 months [126]. Within two weeks of her last transfusion, the patient developed nausea and fever, and an *A. phagocytophilum* infection was diagnosed via increasing anti-*A. phagocytophilum* antibody titers from serum samples taken nine days apart [126]. A course of oral rifampin resolved the symptoms, and the patient gave birth at full-term to a healthy infant. A blood sample from the infant was negative for *A. phagocytophilum* DNA via PCR [126].

There has been only one published case of the vertical transmission of *A. phagocytophilum* [121]. A mother presented with fever (40.6 °C) on the day of delivery and remained febrile for four days after the delivery. She was discharged three days later but returned to the hospital in two days because the infant was sick [121]. The child was admitted with a fever (39.4 °C) and diagnosed with HGA through the presence of morulae in the granulocytes. *A. phagocytophilum* was isolated from a blood draw on the day of admission [121]. The infant started a course of intravenous doxycycline and began to improve within 24 h [121]. It was suspected the infant became infected via vertical transmission, but the precise route (transplacental or via breast milk) was undetermined. 

While no animal studies have been conducted to investigate the rates of *A. phagocytophilum* vertical transmission during acute gestational infection, vertical transmission was noted when an unknowingly pregnant sheep was experimentally infected with *A. phagocytophilum* [41]. The sheep gave birth to a lamb five weeks later, and tissues from the lamb were PCR-positive for *A. phagocytophilum* [41]. Another animal study investigated the consequences of persistent *A. phagocytophilum* infection during pregnancy in sheep [42]. The authors infected virgin ewes with *A. phagocytophilum* four to six weeks prior to mating. Nine ewes became pregnant and delivered twenty lambs, but six died within 2 days [42]. The blood from two of the live lambs was positive for *A. phagocytophilum* DNA, while four of the six dead lambs had multiple tissues test positive [42]. This study provided evidence supporting vertical transmission of *A. phagocytophilum*, although the lack of obtaining an *A. phagocytophilum* isolate from a lamb to confirm the viability is one caveat to this study [42]. The limited number of published perinatal HGA cases and experimental animal studies has hindered our understanding of the vertical transmission mechanisms of *A. phagocytophilum*. 

## 5. Rocky Mountain Spotted Fever

Rocky Mountain spotted fever is a tick-borne disease caused by the obligate intracellular bacterial pathogen *Rickettsia rickettsia* [127,128]. Reports of RMSF cases have been restricted to the Western Hemisphere, primarily in the United States, Mexico, Central America, Brazil, and Colombia [129,130,131,132,133,134,135,136]. *R. rickettsii* is primarily transmitted by the *Ixodidae* tick *Dermacentor variabilis* [127,128]. During the first two weeks following the tick bite, nearly 60–70% of case patients develop fevers, headaches, and rashes, commonly referred to as the “classic clinical triad of disease” [136,137]. The rash may first appear on the wrist and/or ankles of the patients, then spread to the palms and soles and then to the arms, legs, and torso [136]. The case mortality rates of RMSF have been variable between 3% and 44% [129,130,131,138,139]. 

Very little is known regarding the pathogenesis and disease outcomes following RMSF infection during pregnancy [132,140,141,142,143,144,145,146]. RMSF infection during pregnancy can lead to severe adverse maternal outcomes [140,141,142]. Pregnant mothers infected with RMSF experienced respiratory distress leading to intubation and medical intensive care unit stays [141,142,145], amputation of toes and thumbs following necrosis [145], and death [132,140,143]. The outcomes for the fetuses ranged from healthy delivery to fetal death, including stillbirth or abortion [132,140,141,142,143,144,145,146]. One publication failed to detect the presence of *R. rickettsii* in the aborted fetus through immuno-staining [143], while two other studies tested the placenta via immuno-staining or PCR, but did not detect evidence of a *R. rickettsii* infection [142,144]. In the remaining studies, the vertical transmission of *R. rickettsia* was not investigated either because the baby was healthy without signs or symptoms of RMSF, or because it was not considered [132,141,142,146]. Therefore, to our knowledge, there is no evidence of the vertical transmission of *R. rickettsii*. Unfortunately, no animal studies describing *R. rickettsii* pathogenesis, RMSF disease pathology, or maternal and fetal outcomes during pregnancy have been published. 

## 6. Tick-Borne Encephalitis

Tick-borne encephalitis (TBE) is a disease circulating in Northern Europe and Eastern Asia [147,148]. There are approximately 10,000–12,000 annual cases of TBE with the majority reported from Russia [147]. TBE is caused by a Flavivirus named tick-borne encephalitis virus (TBEV), of which there are three subtypes: European, Siberian, and Far Eastern [148,149,150]. TBEV is primarily transmitted by tick species from the *Ixodes* genus; however, it can also be transmitted by the consumption of unpasteurized dairy products [147,150]. While most cases of TBE are asymptomatic, clinical cases can present in two phases [150]. The first viremic phase may induce nonspecific symptoms such as fever, headache, and fatigue, while the second phase may cause meningitis, meningoencephalitis, myelitis, paralysis, radiculitis, and potentially death [150]. Information pertaining to the TBEV disease pathology during pregnancy is sparse because only a few cases have been published. In each case, while the mothers had clinical symptoms ranging from mild to severe, the mothers recovered and gave birth to healthy infants with no neurological or developmental issues during the follow-up periods [151,152,153,154,155]. In one case the mother was hospitalized with aseptic meningoencephalitis, tetraparesis, and polyradiculitis for 85 days, followed by intensive rehabilitation, but the patient gave birth to a healthy boy at term [155]. Even though TBEV is vertically passed in rodents, there was no vertical transmission observed in these reported cases [43,151,152,153,154,155]. However, transmission via milk has been documented in animal models and implicated as a probable route of transmission to infants [156,157]. Epidemiology studies are needed to assess the prevalence and risk of TBEV during pregnancy.

## 7. Crimean-Congo Hemorrhagic Fever

Crimean-Congo hemorrhagic fever (CCHF) is caused by a single-strand negative-sense RNA virus from the *Nairoviridae* family named Crimean-Congo hemorrhagic fever virus (CCHFV) [158,159]. CCHFV is endemic to Africa, the Middle East, Europe, and Asia [158,159]. The virus is primarily transmitted by species of *Hyalomma* ticks from the family *Ixodidae* but can also be transmitted through contact with the bodily fluids of infected patients [158,159]. The signs and symptoms of CCHF include fever, thrombocytopenia, and hemorrhage, with variable case fatality rates up to 40% or higher [160,161,162,163]. While the clinical course of CCHF is well documented, there are few reports describing the outcomes of infection during pregnancy. The only information we have on CCHF during pregnancy comes from case studies and case series; there have not been any large epidemiology studies conducted to date. 

There have been 42 published cases of CCHF during pregnancy. The first described cases of CCHF during pregnancy occurred in the late 1970s and early 1980s in Iraq [164]. All three pregnant females experienced hemorrhage and aborted their fetuses during illness, and two of the three patients passed away [164]. The authors did not investigate the vertical transmission of CCHFV from mother to fetus. There have been 39 additional reports of CCHF during pregnancy that describe both the maternal and fetal outcomes [165,166,167,168,169,170,171,172,173,174,175,176], which were recently part of a systematic review [177]. The mothers commonly reported symptoms such as fever, headache, nausea, and hemorrhage, and in 14 cases the mother died [177]. In 22 of 39 cases, the fetus was aborted or the newborn died shortly after birth [177]. CCHFV infection was detected in deceased newborns or fetuses, demonstrating that CCHFV is vertically transmitted [174,175]. While no animal studies describing the CCHFV pathogenesis, disease pathology, or maternal and fetal outcomes during pregnancy have been published, one study reported vertical transmission of CCHFV in sheep [44]. The authors were unable to isolate the virus from four lambs born 10–40 days post-inoculation of the ewes with CCHFV [44]. More robust animal studies using different species are needed to gain a better understanding of CCHFV vertical transmission and pathogenesis. 

## 8. Babesiosis

Babesiosis is caused by protozoan parasites transmitted by ticks. Only a few out the 100s of *Babesia* species cause disease in humans. The human disease-causing *Babesia* species include *Babesia microti*, *Babesia divergens*, *Babesia duncani*, and *Babesia venatorum* [178]. While babesiosis has been reported in Europe, Asia, Australia, and South America, most of the cases are caused by *B. microti* in the Northeast and Upper Midwest of the United States [178]. *Babesia* species may be transmitted through blood transfusion, but *B. microti* is primarily transmitted to humans by *Ixodes scapularis* [179,180]. Once in a host, *B. microti* invades the erythrocytes and multiplies into daughter cells, which get released and spread to new erythrocytes [178,180]. Common symptoms of *B. microti* infection include fever, fatigue, chills, headache, loss of appetite, and myalgia [178,180]. *B. microti* infections cause severe disease in immunocompromised (HIV, immunosuppressed, or elderly patients) individuals, resulting in possible death [23,181]. 

The risk of severe disease following infection with *Babesia* during pregnancy is not well understood. While the annual numbers of babesiosis reported to the Centers for Disease Control in the United States range from 1000 to 3000 (https://www.cdc.gov/babesiosis/php/data-stats/index.html (accessed on 18 September 2024), there have been less than 15 published cases that document disease progression and maternal and fetal outcomes following *Babesia* infection during pregnancy. The disease severity in pregnant individuals ranged from asymptomatic, to mild, to severe [182,183,184,185,186,187,188,189,190,191]. The reported maternal symptoms included fever, fatigue, chills, headache, joint pain, jaundice, weakness, shortness of breath, cough, respiratory failure, and renal failure [185,186,187,188,189,191]. The disease manifestations in pregnant mothers may resemble Haemolysis, Elevated Liver enzymes, and Low Platelet syndrome (HELLP) [187,188,189,191]. Vertical transmission of *B. microti* has been reported [182,183,184,187,191], and the symptoms in newborns included fever, hyperbilirubinemia, irritability, and loss of appetite [182,183,184]. The symptoms resolved in mothers and newborns following treatment with quinidine and clindamycin with or without a transfusion of packed red blood cells [184,186,187,188,191], or with atovaquone and azithromycin with or without a transfusion of packed red blood cells [182,183,185]. No large-scale epidemiologic studies have been conducted. 

Published animal case reports and studies have helped us understand the impact *Babesia* infection has during pregnancy. The case reports showed *Babesia canis* caused the maternal death of a pregnant feline [192], and *Babesia (Theileria) equi* was vertically transmitted to a fetus and caused an equine abortion [193]. The experimental chronic infection of a canine with *Babesia gibsoni* resulted in a stillbirth and 100% neonatal death 14–39 days post-birth [194]. In this study, a female beagle was infected with 1 × 10^8^ *B. gibsoni* 650 days prior to mating. Following mating, the beagle gave birth to four live pups and one stillborn. Three of the four pups developed parasitemia by day 14 post-birth and all four pups died by day 39 post-birth. The pups were hypothermic, anemic, and showed signs of splenomegaly and hepatomegaly upon death [194]. 

Two studies demonstrated the vertical transmission of *B. microti* in wild-caught rodents [45,46]. The first study was conducted in the Mazury Lake District of northeastern Poland. The authors collected 117 female voles, of which 27 were pregnant and infected with *B. microti* [45]. The vertical transmission of *B. microti* was detected via PCR in 81% of the embryos tested from the pregnant infected voles [45]. The second study collected wild *P. leucopus* from Connecticut and Rhode Island [46]. In total, 74% of embryos tested via qCPR from the infected pregnant *P. leucopus* were infected with *B. microti* [46]. 

Experimental studies performed using pregnant laboratory rodents have begun to delineate vertical transmission and pregnancy outcomes caused by *B. microti* infection. Infecting Wistar rats with *B. microti* three weeks prior to mating impacted the placenta [48]. Compared to the controls, *B. microti* infected rat placentas had elevated erythrocyte adhesion to vascular walls, vacuole formation in the syncytioblasts and cytotrophoblasts, and collagen fiber accumulation in the placental villi [48]. The impact of the experimental infection of *B. microti* prior to mating and during the early and late times of gestation was investigated in the Balb/C mouse model [47,49]. *B. microti* infection seven days prior to mating, on day 4 of gestation, and on day 12 of gestation resulted in either no pregnancy development, resorptions, or stillbirths [47], while infection 40 days prior to mating resulted in live newborns [47]. The vertical transmission of *B. microti* was detected in the offspring following infection on day 12 of gestation and 40 days prior to mating [47]. These animal studies demonstrate that *Babesia* infection can be vertically transmitted from mother to offspring and can severely impact pregnancy. 

## 9. Discussion

The data and reports pertaining to tick-borne disease during pregnancy are very limited, making it difficult to assess if pregnancy increases the risk of adverse outcomes to either the mother or the fetus. It is difficult for physicians to make quick, accurate diagnoses of tick-borne diseases for several reasons. First, most early symptoms associated with tick-borne diseases are nonspecific and include fever, headache, myalgia, and arthralgia. Second, the diagnostic tests for most tick-borne diseases are not designed to detect acute or early infections. Despite these difficulties, the clinical consideration of tick-borne disease during pregnancy is important, especially in locales where the pathogens and vectors are prevalent. 

Another confounding factor is that the conducted studies have resulted in conflicting observations. For example, the case reports of Lyme disease-causing spirochete infection during gestation suggest the disease may be detrimental to perinatal health, but the larger epidemiological support is missing. The adverse outcomes described in the case reports may represent a small percentage of cases that the epidemiological studies were not able to identify due to small cohort sizes that were underpowered [79]. To perform large, robust studies, patient recruitment and enrollment are critical. Work has been done to understand the patient community’s research priorities in hopes of increasing enrollment in future studies [195]. The authors organized focus group interviews with patients who were diagnosed with Lyme disease during pregnancy previously and discovered the patients’ research interests aligned with transmission, testing, treatment, disease presentation, and education [195]. The focus groups also identified concerns about privacy, accessibility, institutional barriers, biological specimen collection, and health risks as potential barriers to enrollment in future studies [195]. This is important information as groups prepare to enroll patients in larger studies to investigate the extent to which Lyme disease affects maternal and perinatal health. These same principles can be applied to investigate the risk of adverse outcomes of the other tick-borne diseases during pregnancy. In future large epidemiology studies, we believe it is critical to evaluate the mechanisms of vertical transmission routes (transplacental, transovarial, or via breastmilk) of each pathogen. 

Pregnancy alters the immune physiology, which can increase the susceptibility to and severity to infectious diseases [24,25,26]. Little is known regarding the immune responses mounted against tick-borne pathogens during pregnancy. Moro et al., found pregnant C3H/HeN mice develop significantly reduced arthritis and inflammation in the tibiotarsal joint compared to non-pregnant females following infection with *B. burgdorferi* [196]. Interestingly, the pathology performed on stillborn or deceased fetuses following maternal Lyme disease revealed no significant inflammation [62,66,67,69,71]. Conversely, multi-organ inflammation was reported in an aborted equine fetus following maternal infection with TBRF spirochetes [102]. Organ pathology has been rarely reported in the cases of fetal death following maternal infection with TBRF spirochetes; however, Fuchs et al., did note inflammatory infiltrates in the meninges of an infant that died within 39 h of birth [103]. It is largely unknown if the altered immune physiology of pregnant females puts them at greater risk to develop severe manifestations of tick-borne diseases. Jongen et al., showed that pregnant individuals have increased TBRF spirochete loads compared to non-pregnant females, but differences in maternal mortality were not observed [101]. Future epidemiological studies should investigate pregnancy as a risk factor that exacerbates tick-borne disease severity in mothers.

Animal models are a powerful tool to elucidate the molecular mechanisms of pathogenesis and disease progression. Unfortunately, few animal studies have investigated the molecular mechanisms of tick-borne disease pathogenesis and disease pathology during pregnancy. The studies that have been conducted to investigate Lyme disease during pregnancy are hard to make generalizable conclusions from because they were not uniform. Each study was performed using different modes of infection in different animal models. Based on the animal studies, *B. burgdorferi* is likely to be vertically transmitted from mother to offspring, although the rate of transmission is unknown. The two studies investigating vertical transmission in wild-caught rodents showed opposite results. One study failed to observe vertical transmission in *P. leucopus* from Massachusetts [38], while the other showed vertical transmission is possible in *M. musculus* and *P. domestics* from a farm in Wisconsin [39]. Interestingly, these studies occurred in two geographically distinct locations (Northeast and Midwest). Perhaps this observed difference is associated with the locally circulating strain of *B. burgdorferi*. The observed differences may be due to the rodent species tested. 

The one animal pregnancy study investigating gestational TBRF provided important insights, including the first experimental evidence of the transplacental transmission of *B. duttonii*; however, there were discrepancies compared to human infection. First, the spirochetemia levels were lower in the infected pregnant mice compared to the infected non-pregnant mice, the opposite of what is observed during human gestational TBRF [40,101]. Second, the authors did not observe perinatal mortality, even though the perinatal mortality can be as high as 43% during human disease [40,101]. Perhaps the route of infection plays a role in the disease progression. This study infected pregnant mice via subcutaneous needle inoculation instead of natural tick transmission. Alternatively, the murine model may have limitations in studying the disease progression because much of the fetal organ development occurs after birth, unlike during human fetal development. Alternatively, the Guinea pig model may present as a better pregnancy model because their pregnancy mimics human placental and fetal development better than mice [197]. Like humans, the Guinea pig placenta circulates elevated levels of progesterone until term, which does not occur in the rodent models [198,199]. Also, fetal Guinea pig brain, heart, and lung development mimics human development better than that of mice. In Guinea pigs and humans, the most rapid synapse formation and myelination occurs in the later stages of gestation, but not until post-birth for other rodents [200,201,202,203]. Similarly, the heart and lung development are more mature at birth in Guinea pigs and humans compared to mice [204,205,206,207,208,209].

Few animal studies have investigated other tick-borne diseases’ pathogenesis or disease pathology during gestational infection; therefore, the continued characterization and development of animal pregnancy models for tick-borne diseases are needed. Once relevant animal pregnancy models are developed for each disease, studies to investigate the molecular mechanisms of vertical transmission and disease pathology can be performed. This will advance our understanding of tick-borne disease infections during pregnancy and accelerate the development of potential intervention strategies. 

Another aspect of gestational tick-borne disease that is rarely considered in case reports, epidemiology, and animal studies is the role of co-infections. Ticks, especially *Ixodes* tick species, can transmit multiple pathogens during a single feeding, including the causative agents of Lyme disease, anaplasmosis, babesiosis, and Powassan virus [210]. Co-infections are rarely described in case reports or epidemiology studies, but may have important, unrealized consequences. Animal pregnancy models will help to investigate the perinatal outcomes from different combinations of tick-borne disease co-infections.

To move the field forward to understand the impact tick-borne diseases have during pregnancy, we need the continued publication of case reports, larger epidemiologic studies, and animal studies using biologically relevant transmission routes in animal models that mimic human disease. Case reports are critical for the continued awareness and consideration of tick-borne infections during pregnancy. Large epidemiological studies are necessary to understand the causal link between tick-borne infections and pregnancy outcomes. Animal studies are powerful ways to delineate the mechanisms of the vertical transmission of tick-borne pathogens and the mechanisms leading to adverse pregnancy outcomes. Currently, these three types of studies have been missing for each tick-borne disease (Table 1). The large-scale epidemiologic studies are lacking for HGA, RMSF, TBE, CCHF, and babesiosis (Table 1). While animal studies have been performed for Lyme disease, TBRF, HGA, TBE, and babesiosis (Table 1), additional robust studies using animals that mimic human pregnancy are warranted to better elucidate the disease outcomes following tick-borne disease during pregnancy. In the event there have been case reports, epidemiologic, and animal studies conducted for a tick-borne disease, the number of published studies remains small, which limits their application. Enhancing our understanding of the impact of tick-borne diseases during pregnancy will lead to improved patient outcomes through the awareness, prompt treatment, and development of novel treatments and/or prevention strategies.

## Figures and Tables

**Table 1:** Publication status of pregnancy case reports, epidemiologic studies, and animal studies for Lyme disease, tick-borne relapsing fever, human granulocytic anaplasmosis, Rocky Mountain spotted fever, tick-borne encephalitis, Crimean-Congo hemorrhagic fever, and babesiosis.

Disease	Case Reports	Epidemiologic Studies	Animal Studies	
Lyme disease	Yes	Yes	Yes C3H/HeN mice [34] Canines [35] Lewis rats [36] Syrian hamsters [37] Wild-caught *P. leucopus* [38] Wild-caught *P. domesticus* [39] Wild-caught *M. musculus* [39]	
Tick-borne relapsing fever	Yes	Yes	Yes C3H/HeN mice [40]	
Human granulocytic anaplasmosis	Yes	No	Yes Sheep [41,42]	
Rocky Mountain spotted fever	Yes	No	No	
Tick-borne encephalitis	Yes	No	Yes Wild-caught red voles [43]	
Crimean-Congo hemorrhagic fever	Yes	No	Yes Sheep [44]	
Babesiosis	Yes	No	Yes Wild-caught *P. leucopus* [45,46,47] Wistar rats [48] Balb/C mice [47,49]	

## References

[1] Mans B.J., de Klerk D., Pienaar R., Latif A.A. (2011). *Nuttalliella namaqua*: A living fossil and closest relative to the ancestral tick lineage: Implications for the evolution of blood-feeding in ticks. PLoS ONE.

[2] Estrada-Pena A., Mangold A.J., Nava S., Venzal J.M., Labruna M., Guglielmone A.A. (2010). A review of the systematics of the tick family Argasidae (Ixodida). Acarologia.

[3] Nava S., Venzal J.M., González-Acuña D., Martins T.F., Guglielmone A.A., Nava S., Venzal J.M., González-Acuña D., Martins T.F., Guglielmone A.A. (2017). Chapter 1—Tick Classification, External Tick Anatomy with a Glossary, and Biological Cycles. Ticks of the Southern Cone of America.

[4] Dutton J.E., Todd J.L. (1905). The nature of tick fever in the eastern part of The Congo Free State. With notes on the distribution and bionomics of the tick. Br. Med. J..

[5] Ricketts H.T. (1991). Some aspects of Rocky Mountain spotted fever as shown by recent investigations. 1909. Rev. Infect. Dis..

[6] Magnavita N., Capitanelli I., Ilesanmi O., Chirico F. (2022). Occupational Lyme disease: A systematic review and meta-analysis. Diagnostics.

[7] Taylor L., Hochuli D.F. (2017). Defining greenspace: Multiple uses across multiple disciplines. Landsc. Urban Plan..

[8] Taylor L., Hochuli D.F. (2015). Creating better cities: How biodiversity and ecosystem functioning enhance urban residents’ wellbeing. Urban Ecosyst..

[9] Hansford K.M., Wheeler B.W., Tschirren B., Medlock J.M. (2022). Questing *Ixodes ricinus* ticks and *Borrelia* spp. in urban green space across Europe: A review. Zoonoses Public Health.

[10] Janzén T., Choudhury F., Hammer M., Petersson M., Dinnétz P. (2024). Ticks—Public health risks in urban green spaces. BMC Public Health.

[11] Cicuttin G.L., De Salvo M.N., Venzal J.M., Nava S. (2019). *Borrelia* spp. in ticks and birds from a protected urban area in Buenos Aires city, Argentina. Ticks Tick-Borne Dis..

[12] Krishnavajhala A., Armstrong B.A., Kneubehl A.R., Gunter S.M., Piccione J., Kim H.J., Ramirez R., Castro-Arellano I., Roachell W., Teel P.D. (2021). Diversity and distribution of the tick-borne relapsing fever spirochete *Borrelia turicatae*. PLoS Negl. Trop. Dis..

[13] VanAcker M.C., Little E.A.H., Molaei G., Bajwa W.I., Diuk-Wasser M.A. (2019). Enhancement of risk for Lyme disease by landscape connectivity, New York, New York, USA. Emerg. Infect. Dis..

[14] Small M., Brennan R.E. (2021). Detection of *Rickettsia amblyommatis* and *Ehrlichia chaffeensis* in *Amblyomma americanum* inhabiting two urban parks in Oklahoma. Vector Borne Zoonotic Dis..

[15] Caixeta B.T., Tolesano-Pascoli G.V., Mundim F.L., Pascoal J.O., Rodrigues V.D.S., Martins M.M., Ramos V.D.N., Torga K., Costa L.F., Miranda V.C. (2024). Survey of *Rickettsia* spp. in ticks (Acari: Ixodidae) infesting opossums (*Didelphis albiventris*) and capybaras (*Hydrochoerus hydrochaeris*) from an urban park in southeastern Brazil. Exp. Appl. Acarol..

[16] Vázquez-Guerrero E., González-Quiroz J.L., Domínguez-López M.L., Kneubehl A.R., Krishnavajhala A., Curtis M.W., Ponce-Mendoza A., Estrada-de Los Santos P., Lopez J.E., Ibarra J.A. (2023). New records of *Ornithodoros turicata* (Ixodida: Argasidae) in rural and urban sites in the Mexican states of Aguascalientes and Zacatecas indicate the potential for tick-borne relapsing fever. Exp. Appl. Acarol..

[17] Ritchie H., Samborska V., Roser M. Urbanization. https://ourworldindata.org/urbanization.

[18] World Urbanization Prospects: The 2018 Revision. https://population.un.org/wup/Download/.

[19] Bermúdez S.E., Castillo E., Pohlenz T.D., Kneubehl A., Krishnavajhala A., Domínguez L., Suárez A., López J.E. (2017). New records of *Ornithodoros puertoricensis* Fox 1947 (Ixodida: Argasidae) parasitizing humans in rural and urban dwellings, Panama. Ticks Tick-Borne Dis..

[20] Vázquez-Guerrero E., Kneubehl A.R., Pellegrini-Hernández P., González-Quiroz J.L., Domínguez-López M.L., Krishnavajhala A., Estrada-de Los Santos P., Ibarra J.A., Lopez J.E. (2024). *Borrelia turicatae* from ticks in peridomestic setting, Camayeca, Mexico. Emerg. Infect. Dis..

[21] Godfrey E.R., Randolph S.E. (2011). Economic downturn results in tick-borne disease upsurge. Parasites Vectors.

[22] Brouqui P., Stein A., Dupont H.T., Gallian P., Badiaga S., Rolain J.M., Mege J.L., La Scola B., Berbis P., Raoult D. (2005). Ectoparasitism and vector-borne diseases in 930 homeless people from Marseilles. Medicine.

[23] Krause P.J., Gewurz B.E., Hill D., Marty F.M., Vannier E., Foppa I.M., Furman R.R., Neuhaus E., Skowron G., Gupta S. (2008). Persistent and relapsing babesiosis in immunocompromised patients. Clin. Infect. Dis..

[24] Abu-Raya B., Michalski C., Sadarangani M., Lavoie P.M. (2020). Maternal immunological adaptation during normal pregnancy. Front. Immunol..

[25] Robinson D.P., Klein S.L. (2012). Pregnancy and pregnancy-associated hormones alter immune responses and disease pathogenesis. Horm. Behav..

[26] Kourtis A.P., Read J.S., Jamieson D.J. (2014). Pregnancy and infection. N. Engl. J. Med..

[27] Siston A.M., Rasmussen S.A., Honein M.A., Fry A.M., Seib K., Callaghan W.M., Louie J., Doyle T.J., Crockett M., Lynfield R. (2010). Pandemic 2009 influenza A(H1N1) virus illness among pregnant women in the United States. JAMA.

[28] Mosby L.G., Rasmussen S.A., Jamieson D.J. (2011). 2009 pandemic influenza A (H1N1) in pregnancy: A systematic review of the literature. Am. J. Obstet. Gynecol..

[29] Desai M., ter Kuile F.O., Nosten F., McGready R., Asamoa K., Brabin B., Newman R.D. (2007). Epidemiology and burden of malaria in pregnancy. Lancet Infect. Dis..

[30] Ali M.E., Albar H.M. (1997). Measles in pregnancy: Maternal morbidity and perinatal outcome. Int. J. Gynaecol. Obstet..

[31] Siegel M., Fuerst H.T. (1966). Low birth weight and maternal virus diseases: A prospective study of rubella, measles, mumps, chickenpox, and hepatitis. JAMA.

[32] Atmar R.L., Englund J.A., Hammill H. (1992). Complications of measles during pregnancy. Clin. Infect. Dis..

[33] Shperling R.B., Yogev Y. (2022). Adverse outcomes of measles infection during pregnancy and in the perinatal period. J. Matern. Fetal Neonatal Med..

[34] Silver R.M., Yang L., Daynes R.A., Branch D.W., Salafia C.M., Weis J.J. (1995). Fetal outcome in murine Lyme disease. Infect. Immun..

[35] Gustafson J.M., Burgess E.C., Wachal M.D., Steinberg H. (1993). Intrauterine transmission of *Borrelia burgdorferi* in dogs. Am. J. Vet. Res..

[36] Moody K.D., Barthold S.W. (1991). Relative infectivity of *Borrelia burgdorferi* in Lewis rats by various routes of inoculation. Am. J. Trop. Med. Hyg..

[37] Woodrum J.E., Oliver J.H. (1999). Investigation of venereal, transplacental, and contact transmission of the Lyme disease spirochete, *Borrelia burgdorferi*, in Syrian hamsters. J. Parasitol..

[38] Mather T.N., Telford S.R., Adler G.H. (1991). Absence of transplacental transmission of Lyme disease spirochetes from reservoir mice (*Peromyscus leucopus*) to their offspring. J. Infect. Dis..

[39] Burgess E.C., Wachal M.D., Cleven T.D. (1993). *Borrelia burgdorferi* infection in dairy cows, rodents, and birds from four Wisconsin dairy farms. Vet. Microbiol..

[40] Larsson C., Andersson M., Guo B.P., Nordstrand A., Hägerstrand I., Carlsson S., Bergström S. (2006). Complications of pregnancy and transplacental transmission of relapsing-fever borreliosis. J. Infect. Dis..

[41] Reppert E., Galindo R.C., Breshears M.A., Kocan K.M., Blouin E.F., de la Fuente J. (2013). Demonstration of transplacental transmission of a human isolate of *Anaplasma phagocytophilum* in an experimentally infected sheep. Transbound. Emerg. Dis..

[42] Stuen S., Okstad W., Sagen A.M. (2018). Intrauterine transmission of *Anaplasma phagocytophilum* in persistently infected lambs. Vet. Sci..

[43] Bakhvalova V.N., Potapova O.F., Panov V.V., Morozova O.V. (2009). Vertical transmission of tick-borne encephalitis virus between generations of adapted reservoir small rodents. Virus Res..

[44] Gonzalez J.P., Camicas J.L., Cornet J.P., Wilson M.L. (1998). Biological and clinical responses of West African sheep to Crimean-Congo haemorrhagic fever virus experimental infection. Res. Virol..

[45] Tołkacz K., Bednarska M., Alsarraf M., Dwużnik D., Grzybek M., Welc-Falęciak R., Behnke J.M., Bajer A. (2017). Prevalence, genetic identity and vertical transmission of *Babesia microti* in three naturally infected species of vole, *Microtus* spp. (Cricetidae). Parasit. Vectors.

[46] Tufts D.M., Diuk-Wasser M.A. (2018). Transplacental transmission of tick-borne *Babesia microti* in its natural host *Peromyscus leucopus*. Parasit. Vectors.

[47] Tołkacz K., Rodo A., Wdowiarska A., Bajer A., Bednarska M. (2021). Impact of *Babesia microti* infection on the initiation and course of pregnancy in BALB/c mice. Parasit. Vectors.

[48] Jasik K.P., Kleczka A., Franielczyk A. (2024). Histopathological aspects of the influence of *Babesia microti* on the placentas of infected female rats. Vet. Sci..

[49] Bednarska M., Bajer A., Drozdowska A., Mierzejewska E.J., Tolkacz K., Welc-Falęciak R. (2015). Vertical transmission of *Babesia* microti in BALB/c mice: Preliminary report. PLoS ONE.

[50] Burn L., Tran T.M.P., Pilz A., Vyse A., Fletcher M.A., Angulo F.J., Gessner B.D., Moïsi J.C., Jodar L., Stark J.H. (2023). Incidence of Lyme Borreliosis in Europe from National Surveillance Systems (2005–2020). Vector Borne Zoonotic Dis..

[51] Cardenas-de la Garza J.A., De la Cruz-Valadez E., Ocampo-Candiani J., Welsh O. (2019). Clinical spectrum of Lyme disease. Eur. J. Clin. Microbiol. Infect. Dis..

[52] Gerber M.A., Shapiro E.D., Burke G.S., Parcells V.J., Bell G.L. (1996). Lyme disease in children in southeastern Connecticut. Pediatric Lyme Disease Study Group. N. Engl. J. Med..

[53] Krause P.J., Telford S.R., Spielman A., Sikand V., Ryan R., Christianson D., Burke G., Brassard P., Pollack R., Peck J. (1996). Concurrent Lyme disease and babesiosis. Evidence for increased severity and duration of illness. JAMA.

[54] Hu L.T. (2016). Lyme Disease. Ann. Intern. Med..

[55] Steere A.C. (1989). Lyme disease. N. Engl. J. Med..

[56] Garcia-Monco J.C., Benach J.L. (2019). Lyme neuroborreliosis: Clinical outcomes, controversy, pathogenesis, and polymicrobial infections. Ann. Neurol..

[57] Arvikar S.L., Steere A.C. (2022). Lyme arthritis. Infect. Dis. Clin. N. Am..

[58] Centers for Disease Control (1985). Update: Lyme disease and cases occurring during pregnancy—United States. MMWR Morb. Mortal. Wkly. Rep..

[59] Grandsaerd M.J., Meulenbroeks A.A. (2000). Lyme borreliosis as a cause of facial palsy during pregnancy. Eur. J. Obstet. Gynecol. Reprod. Biol..

[60] Maraspin V., Ružić-Sabljić E., Pleterski-Rigler D., Strle F. (2011). Pregnant women with erythema migrans and isolation of borreliae from blood: Course and outcome after treatment with ceftriaxone. Diagn. Microbiol. Infect. Dis..

[61] Schaumann R., Fingerle V., Buchholz K., Spencker F.B., Rodloff A.C. (1999). Facial palsy caused by *Borrelia* infection in a twin pregnancy in an area of nonendemicity. Clin. Infect. Dis..

[62] Mikkelsen A.L., Palle C. (1987). Lyme disease during pregnancy. Acta Obstet. Gynecol. Scand..

[63] Trevisan G., Ruscio M., di Meo N., Nan K., Cinco M., Trevisini S., Forgione P., Bonin S. (2022). Case Report: Lyme Borreliosis and Pregnancy—Our Experience. Front. Med..

[64] Trevisan G., Stinco G., Cinco M. (1997). Neonatal skin lesions due to a spirochetal infection: A case of congenital Lyme borreliosis?. Int. J. Dermatol..

[65] Pavia C.S., Plummer M.M., Varantsova A. (2024). An unusual case of serologically confirmed post-partum Lyme disease following an asymptomatic *Borrelia burgdorferi* infection acquired during pregnancy and lacking vertical transmission in utero. Pathogens.

[66] Schlesinger P.A., Duray P.H., Burke B.A., Steere A.C., Stillman M.T. (1985). Maternal-fetal transmission of the Lyme disease spirochete, *Borrelia burgdorferi*. Ann. Intern. Med..

[67] MacDonald A.B., Benach J.L., Burgdorfer W. (1987). Stillbirth following maternal Lyme disease. N. Y State J. Med..

[68] Moniuszko A., Czupryna P., Pancewicz S., Kondrusik M., Penza P., Zajkowska J. (2012). Borrelial lymphocytoma—A case report of a pregnant woman. Ticks Tick-Borne Dis..

[69] Weber K., Bratzke H.-J., Neubert U., Wilske B., Duray P.H. (1988). *Borrelia burgdorferi* in a newborn despite oral penicillin for Lyme borreliosis during pregnancy. Pediatr. Infect. Dis. J..

[70] Courville J.M., Czuzoj-Shulman N., Spence A.R., Abenhaim H.A. (2024). Obstetrical and neonatal outcomes in women with gestational Lyme disease. Int. J. Gynaecol. Obstet..

[71] Markowitz L.E., Steere A.C., Benach J.L., Slade J.D., Broome C.V. (1986). Lyme disease during pregnancy. JAMA.

[72] Gardner T., Remington J., Klein J. (2001). Lyme disease. Infectious Diseases of the Fetus and Newborn Infant.

[73] McCormick D.W., Brown C.M., Bjork J., Cervantes K., Esponda-Morrison B., Garrett J., Kwit N., Mathewson A., McGinnis C., Notarangelo M. (2023). Characteristics of hard tick relapsing fever caused by *Borrelia miyamotoi*, United States, 2013–2019. Emerg. Infect. Dis..

[74] Kingry L.C., Anacker M., Pritt B., Bjork J., Respicio-Kingry L., Liu G., Sheldon S., Boxrud D., Strain A., Oatman S. (2018). Surveillance for and discovery of *Borrelia* species in US patients suspected of tickborne illness. Clin. Infect. Dis..

[75] Molloy P.J., Weeks K.E., Todd B., Wormser G.P. (2018). Seroreactivity to the C6 peptide in *Borrelia miyamotoi* infections occurring in the Northeastern United States. Clin. Infect. Dis..

[76] Gettings J.R., Lopez J.E., Krishnavahjala A., Armstrong B.A., Thompson A.T., Yabsley M.J. (2019). Antibodies to *Borrelia turicatae* in experimentally infected dogs cross-react with *Borrelia burgdorferi* serologic assays. J. Clin. Microbiol..

[77] Rath P.M., Rögler G., Schönberg A., Pohle H.D., Fehrenbach F.J. (1992). Relapsing fever and its serological discrimination from Lyme borreliosis. Infection.

[78] Figueroa R., Bracero L.A., Aguero-Rosenfeld M., Beneck D., Coleman J., Schwartz I. (2010). Confirmation of *Borrelia burgdorferi* spirochetes by polymerase chain reaction in placentas of women with reactive serology for Lyme antibodies. Gynecol. Obstet. Investig..

[79] Strobino B.A., Williams C.L., Abid S., Chalson R., Spierling P. (1993). Lyme disease and pregnancy outcome: A prospective study of two thousand prenatal patients. Am. J. Obstet. Gynecol..

[80] Strobino B., Abid S., Gewitz M. (1999). Maternal Lyme disease and congenital heart disease: A case-control study in an endemic area. Am. J. Obstet. Gynecol..

[81] Williams C.L., Strobino B., Weinstein A., Spierling P., Medici F. (1995). Maternal Lyme disease and congenital malformations: A cord blood serosurvey in endemic and control areas. Paediatr. Perinat. Epidemiol..

[82] Leavey K., MacKenzie R.K., Faber S., Lloyd V.K., Mao C., Wills M.K.B., Boucoiran I., Cates E.C., Omar A., Marquez O. (2022). Lyme borreliosis in pregnancy and associations with parent and offspring health outcomes: An international cross-sectional survey. Front. Med..

[83] Waddell L.A., Greig J., Lindsay L.R., Hinckley A.F., Ogden N.H. (2018). A systematic review on the impact of gestational Lyme disease in humans on the fetus and newborn. PLoS ONE.

[84] Tang T., Zhu Y., Zhang Y.Y., Chen J.J., Tian J.B., Xu Q., Jiang B.G., Wang G.L., Golding N., Mehlman M.L. (2024). The global distribution and the risk prediction of relapsing fever group *Borrelia*: A data review with modelling analysis. Lancet Microbe.

[85] Cutler S.J. (2015). Relapsing fever borreliae: A global review. Clin. Lab. Med..

[86] Barstad P.A., Coligan J.E., Raum M.G., Barbour A.G. (1985). Variable major proteins of *Borrelia hermsii*. Epitope mapping and partial sequence analysis of CNBr peptides. J. Exp. Med..

[87] Plasterk R.H.A., Simon M.I., Barbour A.G. (1985). Transposition of structural genes to an expression sequence on a linear plasmid causes antigenic variation in the bacterium *Borrelia hermsii*. Nature.

[88] Meier J.T., Simon M.I., Barbour A.G. (1985). Antigenic variation is associated with DNA rearrangements in a relapsing fever *Borrelia*. Cell.

[89] Lopez J., Hovius J.W., Bergström S. (2021). Pathogenesis of relapsing fever. Curr. Issues Mol. Biol..

[90] Saint Girons I., Barbour A.G. (1991). Antigenic variation in *Borrelia*. Res. Microbiol..

[91] Barbour A.G. (1990). Antigenic variation of a relapsing fever *Borrelia* species. Annu. Rev. Microbiol..

[92] Barbour A.G., Burman N., Carter C.J., Kitten T., Bergström S. (1991). Variable antigen genes of the relapsing fever agent *Borrelia hermsii* are activated by promoter addition. Mol. Microbiol..

[93] Dai Q., Restrepo B.I., Porcella S.F., Raffel S.J., Schwan T.G., Barbour A.G. (2006). Antigenic variation by *Borrelia hermsii* occurs through recombination between extragenic repetitive elements on linear plasmids. Mol. Microbiol..

[94] Kneubehl A.R., Lopez J.E. (2023). Comparative genomics analysis of three conserved plasmid families in the Western Hemisphere soft tick-borne relapsing fever borreliae provides insight into variation in genome structure and antigenic variation systems. Microbiol. Spectr..

[95] Dworkin M.S., Schwan T.G., Anderson D.E. (2002). Tick-borne relapsing fever in North America. Med. Clin. N. Am..

[96] Southern P.M., Sanford J.P. (1969). Relapsing fever: A clinical and microbiological review. Medicine.

[97] Assous M.V., Wilamowski A. (2009). Relapsing fever borreliosis in Eurasia—forgotten, but certainly not gone!. Clin. Microbiol. Infect..

[98] Davis H., Vincent J.M., Lynch J. (2002). Tick-borne relapsing fever caused by *Borrelia turicatae*. Pediatr. Infect. Dis. J..

[99] Rawlings J.A. (1995). An overview of tick-borne relapsing fever with emphasis on outbreaks in Texas. Tex. Med..

[100] Vial L., Diatta G., Tall A., Ba E.H., Bouganali H., Durand P., Sokhna C., Rogier C., Renaud F., Trape J.F. (2006). Incidence of tick-borne relapsing fever in west Africa: Longitudinal study. Lancet.

[101] Jongen V.H., van Roosmalen J., Tiems J., Van Holten J., Wetsteyn J.C. (1997). Tick-borne relapsing fever and pregnancy outcome in rural Tanzania. Acta Obstet. Gynecol. Scand..

[102] Walker R.L., Read D.H., Hayes D.C., Nordhausen R.W. (2002). Equine abortion associated with the *Borrelia parkeri*-*B. turicatae* tick-borne relapsing fever spirochete group. J. Clin. Microbiol..

[103] Fuchs P.C., Oyama A.A. (1969). Neonatal relapsing fever due to transplacental transmission of *Borrelia*. JAMA.

[104] Brasseur D. (1985). Tick-borne relapsing fever in a premature infant. Ann. Trop. Paediatr..

[105] Dupont H.T., La Scola B., Williams R., Raoult D. (1997). A focus of tick-borne relapsing fever in Southern Zaire. Clin. Infect. Dis..

[106] Rustenhoven-Spaan I., Melkert P., Nelissen E., van Roosmalen J., Stekelenburg J. (2013). Maternal mortality in a rural Tanzanian hospital: Fatal Jarisch-Herxheimer reaction in a case of relapsing fever in pregnancy. Trop. Dr..

[107] Melkert P.W. (1988). Relapsing fever in pregnancy: Analysis of high-risk factors. Br. J. Obstet. Gynaecol..

[108] Mahram M., Ghavami M.B. (2009). Congenital tick-borne relapsing fever: Report of a case with transplacental transmission in the Islamic Republic of Iran. East. Mediterr. Health J..

[109] CDC (2012). Tickborne relapsing fever in a mother and newborn child—Colorado, 2011. MMWR Morb. Mortal. Wkly. Rep..

[110] Lam J.C., Larios O.E., Parkins M.D., Vaughan S.D. (2020). A case of tick-borne relapsing fever in pregnancy. Can. Commun. Dis. Rep..

[111] Hussein H., Showler A., Tan D.H. (2014). Tick-borne relapsing fever in pregnancy. CMAJ.

[112] Malison M.D. (1979). Relapsing fever. JAMA.

[113] Davis R.D., Burke J.P., Wright L.J. (1992). Relapsing fever associated with ARDS in a parturient woman. A case report and review of the literature. Chest.

[114] Steenbarger J.R. (1982). Congenital tick-borne relapsing fever: Report of a case with first documentation of transplacental transmission. Birth Defects Orig. Artic. Ser..

[115] Stuen S., Granquist E.G., Silaghi C. (2013). *Anaplasma phagocytophilum*—A widespread multi-host pathogen with highly adaptive strategies. Front. Cell. Infect. Microbiol..

[116] Dumler J.S., Choi K.S., Garcia-Garcia J.C., Barat N.S., Scorpio D.G., Garyu J.W., Grab D.J., Bakken J.S. (2005). Human granulocytic anaplasmosis and *Anaplasma phagocytophilum*. Emerg. Infect. Dis..

[117] Dumic I., Jevtic D., Veselinovic M., Nordstrom C.W., Jovanovic M., Mogulla V., Veselinovic E.M., Hudson A., Simeunovic G., Petcu E. (2022). Human granulocytic anaplasmosis-A systematic review of published cases. Microorganisms.

[118] (2016). Tickborne fever associated with abortion outbreak in dairy cows. Vet. Rec..

[119] Chochlakis D., Giadinis N., Petridou E., Filioussis G., Tselentis Y., Psaroulaki A., Ioannidou E., Papanikolopoulou V., Karatzias H. (2020). Molecular evidence of *Anaplasma phagocytophilum* in aborted goat fetuses and placenta. Vet. Ital..

[120] Henker L.C., Lorenzett M.P., Fagundes-Moreira R., Dalto A.G.C., Sonne L., Driemeier D., Soares J.F., Pavarini S.P. (2020). Bovine abortion, stillbirth and neonatal death associated with *Babesia bovis* and *Anaplasma* sp. infections in southern Brazil. Ticks Tick-Borne Dis..

[121] Horowitz H.W., Kilchevsky E., Haber S., Aguero-Rosenfeld M., Kranwinkel R., James E.K., Wong S.J., Chu F., Liveris D., Schwartz I. (1998). Perinatal transmission of the agent of human ganulocytic erlichiosis. N. Engl. J. Med..

[122] Dhand A., Nadelman R.B., Aguero-Rosenfeld M., Haddad F.A., Stokes D.P., Horowitz H.W. (2007). Human granulocytic anaplasmosis during pregnancy: Case series and literature review. Clin. Infect. Dis..

[123] Casau N.C., Hewins M.E., Zaleznik D.F. (2002). Treatment of human granulocytic ehrlichiosis during pregnancy and risk of perinatal transmission. Scand. J. Infect. Dis..

[124] Buitrago M.I., Ijdo J.W., Rinaudo P., Simon H., Copel J., Gadbaw J., Heimer R., Fikrig E., Bia F.J. (1998). Human granulocytic ehrlichiosis during pregnancy treated successfully with rifampin. Clin. Infect. Dis..

[125] Qasba N., Shamshirsaz A.A., Feder H.M., Campbell W.A., Egan J.F., Shamshirsaz A.A. (2011). A case report of human granulocytic anaplasmosis (ehrlichiosis) in pregnancy and a literature review of tick-borne diseases in the United States during pregnancy. Obstet. Gynecol. Surv..

[126] Shields K., Cumming M., Rios J., Wong M.T., Zwicker J.I., Stramer S.L., Alonso C.D. (2015). Transfusion-associated *Anaplasma phagocytophilum* infection in a pregnant patient with thalassemia trait: A case report. Transfusion.

[127] Dantas-Torres F. (2007). Rocky Mountain spotted fever. Lancet Infect. Dis..

[128] Kim H.K. (2022). Rickettsia-host-tick interactions: Knowledge advances and gaps. Infect. Immun..

[129] Jay R., Armstrong P.A. (2020). Clinical characteristics of Rocky Mountain spotted fever in the United States: A literature review. J. Vector Borne Dis..

[130] Kjemtrup A.M., Padgett K., Paddock C.D., Messenger S., Hacker J.K., Feiszli T., Melgar M., Metzger M.E., Hu R., Kramer V.L. (2022). A forty-year review of Rocky Mountain spotted fever cases in California shows clinical and epidemiologic changes. PLOS Neglected Trop. Dis..

[131] Zazueta O.E., Armstrong P.A., Márquez-Elguea A., Hernández Milán N.S., Peterson A.E., Ovalle-Marroquín D.F., Fierro M., Arroyo-Machado R., Rodriguez-Lomeli M., Trejo-Dozal G. (2021). Rocky mountain spotted fever in a large metropolitan center, Mexico-United States border, 2009–2019. Emerg. Infect. Dis..

[132] Tribaldos M., Zaldivar Y., Bermudez S., Samudio F., Mendoza Y., Martinez A.A., Villalobos R., Eremeeva M.E., Paddock C.D., Page K. (2011). Rocky Mountain spotted fever in Panama: A cluster description. J. Infect. Dev. Ctries..

[133] de Lemos E.R., Machado R.D., Coura J.R. (1994). Rocky Mountain spotted fever in an endemic area in Minas Gerais, Brazil. Mem. Inst. Oswaldo Cruz.

[134] Hidalgo M., Orejuela L., Fuya P., Carrillo P., Hernandez J., Parra E., Keng C., Small M., Olano J.P., Bouyer D. (2007). Rocky Mountain spotted fever, Colombia. Emerg. Infect. Dis..

[135] Hidalgo M., Miranda J., Heredia D., Zambrano P., Vesga J.F., Lizarazo D., Mattar S., Valbuena G. (2011). Outbreak of Rocky Mountain spotted fever in Córdoba, Colombia. Mem. Inst. Oswaldo Cruz.

[136] Thorner A.R., Walker D.H., Petri W.A. (1998). Rocky mountain spotted fever. Clin. Infect. Dis..

[137] Nathavitharana R.R., Mitty J.A. (2015). Diseases from North America: Focus on tick-borne infections. Clin. Med..

[138] Regan J.J., Traeger M.S., Humpherys D., Mahoney D.L., Martinez M., Emerson G.L., Tack D.M., Geissler A., Yasmin S., Lawson R. (2015). Risk factors for fatal outcome from rocky mountain spotted Fever in a highly endemic area-Arizona, 2002–2011. Clin. Infect. Dis..

[139] Álvarez-López D.I., Ochoa-Mora E., Nichols Heitman K., Binder A.M., Álvarez-Hernández G., Armstrong P.A. (2021). Epidemiology and clinical features of rocky mountain spotted fever from enhanced surveillance, Sonora, Mexico: 2015–2018. Am. J. Trop. Med. Hyg..

[140] Ponce Nájera E., Lozano Lazcano V., Ploneda González C., Montoya Hinojosa M., González Oropeza D. (2024). Case report: Fatal rickettsiosis in pregnancy. Am. J. Trop. Med. Hyg..

[141] Stallings S.P. (2001). Rocky Mountain spotted fever and pregnancy: A case report and review of the literature. Obstet. Gynecol. Surv..

[142] Wu J., Dotters-Katz S.K., Varvoutis M. (2024). Atypical presentation of rocky mountain spotted fever in pregnancy. AJP Rep..

[143] de Lemos E.R., Alvarenga F.B., Cintra M.L., Ramos M.C., Paddock C.D., Ferebee T.L., Zaki S.R., Ferreira F.C., Ravagnani R.C., Machado R.D. (2001). Spotted fever in Brazil: A seroepidemiological study and description of clinical cases in an endemic area in the state of São Paulo. Am. J. Trop. Med. Hyg..

[144] Markley K.C., Levine A.B., Chan Y. (1998). Rocky mountain spotted fever in pregnancy. Obstet. Gynecol..

[145] Licona-Enriquez J.D., Delgado-de la Mora J., Paddock C.D., Ramirez-Rodriguez C.A., Candia-Plata M.D.C., Hernández G. (2017). Rocky mountain spotted fever and pregnancy: Four cases from Sonora, Mexico. Am. J. Trop. Med. Hyg..

[146] Gallis H.A., Agner R.C., Painter C.J. (1984). Rocky Mountain spotted fever in pregnancy. N. Carol. Med. J..

[147] Chiffi G., Grandgirard D., Leib S.L., Chrdle A., Růžek D. (2023). Tick-borne encephalitis: A comprehensive review of the epidemiology, virology, and clinical picture. Rev. Med. Virol..

[148] Kwasnik M., Rola J., Rozek W. (2023). Tick-borne encephalitis-Review of the current status. J. Clin. Med..

[149] Ecker M., Allison S.L., Meixner T., Heinz F.X. (1999). Sequence analysis and genetic classification of tick-borne encephalitis viruses from Europe and Asia. J. Gen. Virol..

[150] Ruzek D., Avšič Županc T., Borde J., Chrdle A., Eyer L., Karganova G., Kholodilov I., Knap N., Kozlovskaya L., Matveev A. (2019). Tick-borne encephalitis in Europe and Russia: Review of pathogenesis, clinical features, therapy, and vaccines. Antivir. Res..

[151] Kepková M. (2023). Tick-borne encephalitis in pregnancy. Ceska Gynekol..

[152] Weinmayr L.M., Kanz D., Eckenweiler M., Bormann T., Huzly D., Bardutzky J., Harloff A. (2020). Acute tick-borne encephalitis during pregnancy—An alarming combination. Ticks Tick-Borne Dis..

[153] Bjonholm E., Soderholm S., Stephansson O., Askling H.H. (2022). Tick-borne encephalitis in pregnant women: A mini narrative review. New Microbes New Infect..

[154] Divé I., Veje M., Dobler G., Bergström T., Buxmann H., Paul B., Louwen F., Berger A., Jahnke K., Strzelczyk A. (2020). Tick-borne encephalitis virus (TBEV) infection in pregnancy: Absence of virus transmission to the fetuses despite severe maternal disease—A case study. Ticks Tick-Borne Dis..

[155] Velay A., Janssen-Langenstein R., Kremer S., Laugel E., Lutz M., Pierson A.L., Wendling M.J., Schneider F., Fafi-Kremer S. (2023). Tick-borne encephalitis in pregnant woman and long-term sequelae. Emerg. Infect. Dis..

[156] Dabas R., Sharma N., Taksande A.B., Prasad R., Munjewar P.K., Wanjari M.B. (2023). Breast milk: A potential route of tick-borne encephalitis virus transmission from mother to infant. Cureus.

[157] Kerlik J., Avdičová M., Musilová M., Bérešová J., Mezencev R. (2022). Breast milk as route of tick-borne encephalitis virus transmission from mother to infant. Emerg. Infect. Dis..

[158] Fillâtre P., Revest M., Tattevin P. (2019). Crimean-Congo hemorrhagic fever: An update. Med. Mal. Infect..

[159] Hawman D.W., Feldmann H. (2023). Crimean-Congo haemorrhagic fever virus. Nat. Rev. Microbiol..

[160] Lorenzo Juanes H.M., Carbonell C., Sendra B.F., López-Bernus A., Bahamonde A., Orfao A., Lista C.V., Ledesma M.S., Negredo A.I., Rodríguez-Alonso B. (2023). Crimean-Congo hemorrhagic fever, Spain, 2013–2021. Emerg. Infect. Dis..

[161] Ergönül Ö., Çelikbaş A., Dokuzoğuz B., Eren Ş., Baykam N., Esener H. (2004). Characteristics of patients with Crimean-Congo hemorrhagic fever in a recent outbreak in Turkey and impact of oral ribavirin therapy. Clin. Infect. Dis..

[162] Ahmeti S., Berisha L., Halili B., Ahmeti F., von Possel R., Thomé-Bolduan C., Michel A., Priesnitz S., Reisinger E.C., Günther S. (2019). Crimean-Congo hemorrhagic fever, Kosovo, 2013–2016. Emerg. Infect. Dis..

[163] Keshtkar-Jahromi M., Sajadi M.M., Ansari H., Mardani M., Holakouie-Naieni K. (2013). Crimean-Congo hemorrhagic fever in Iran. Antivir. Res..

[164] Al-Tikriti S.K., Al-Ani F., Jurji F.J., Tantawi H., Al-Moslih M., Al-Janabi N., Mahmud M.I., Al-Bana A., Habib H., Al-Munthri H. (1981). Congo/Crimean haemorrhagic fever in Iraq. Bull. World Health Organ..

[165] Nabeth P., Cheikh D.O., Lo B., Faye O., Vall I.O.M., Niang M., Wague B., Diop D., Diallo M., Diallo B. (2004). Crimean-Congo Hemorrhagic Fever, Mauritania. Emerg. Infect. Dis. J..

[166] Naderi H.R., Sarvghad M.R., Bojdy A., Hadizadeh M.R., Sadeghi R., Sheybani F. (2011). Nosocomial outbreak of Crimean-Congo haemorrhagic fever. Epidemiol. Infect..

[167] Pshenichnaya N.Y., Nenadskaya S.A. (2015). Probable Crimean-Congo hemorrhagic fever virus transmission occurred after aerosol-generating medical procedures in Russia: Nosocomial cluster. Int. J. Infect. Dis..

[168] Mardani M., Namazee N. (2013). Close contact precautions could prevent an outbreak of crimean-congo hemorrhagic Fever: A case series report from southern part of tehran. Int. J. Prev. Med..

[169] Duygu F., Cicek A.C., Kaya T. (2015). Crimean-Congo hemorrhagic fever and pregnancy: Two cases. J. Microbiol. Infect. Dis..

[170] Mood B.S., Mardani M., Metanat M. (2007). Clinical manifestations, laboratory findings and clinical outcome in 6 pregnant women with Crimean-Congo hemorrhagic fever *Iran*. J. Clin. Infect. Dis..

[171] Dizbay M., Aktas F., Gaygisiz U., Ozger H.S., Ozdemir K. (2009). Crimean-Congo hemorrhagic fever treated with ribavirin in a pregnant woman. J. Infect..

[172] Gozel M.G., Elaldi N., Engin A., Akkar O.B., Bolat F., Celik C. (2014). Favorable outcomes for both mother and baby are possible in pregnant women with Crimean-Congo hemorrhagic fever disease: A case series and literature review. Gynecol. Obstet. Investig..

[173] Ünlüsoy-Aksu A., Havali C., Tapisiz A., Aktaş F., Ezgu F. (2014). Crimean-congo haemorrhagic fever in pregnancy and in newborn: A case with a unique clinical course. J. Obstet. Gynaecol..

[174] Ergonul O., Celikbas A., Yildirim U., Zenciroglu A., Erdogan D., Ziraman I., Saracoglu F., Demirel N., Cakmak O., Dokuzoguz B. (2010). Pregnancy and Crimean-Congo haemorrhagic fever. Clin. Microbiol. Infect..

[175] Dedkov V.G., Shchelkanov M.Y., Bushkieva B.T., Rudenko T.A., Kurdyukova O.V., Galkina I.V., Sapotsky M.V., Blinova E.A., Dzhambinov S.D., Shipulin G.A. (2017). A neonatal death associated with Crimean-Congo hemorrhagic fever (Republic of Kalmykia, Russia, June 2016). Antivir. Res.

[176] Aydemir O., Erdeve O., Oguz S.S., Dilmen U. (2010). A healthy newborn born to a mother with Crimean-Congo hemorrhagic fever: Is there protection from transplacental transmission?. Int. J. Infect. Dis..

[177] Pshenichnaya N.Y., Leblebicioglu H., Bozkurt I., Sannikova I.V., Abuova G.N., Zhuravlev A.S., Barut S., Shermetova M.B., Fletcher T.E. (2017). Crimean-Congo hemorrhagic fever in pregnancy: A systematic review and case series from Russia, Kazakhstan and Turkey. Int. J. Infect. Dis..

[178] Krause P.J. (2019). Human babesiosis. Int. J. Parasitol..

[179] Spielman A. (1976). Human Babesiosis on Nantucket Island: Transmission by nymphal Ixodes ticks. Am. J. Trop. Med. Hyg..

[180] Vannier E.G., Diuk-Wasser M.A., Ben Mamoun C., Krause P.J. (2015). Babesiosis. Infect. Dis. Clin. N. Am..

[181] Meldrum S.C., Birkhead G.S., White D.J., Benach J.L., Morse D.L. (1992). Human babesiosis in New York State: An epidemiological description of 136 cases. Clin. Infect. Dis..

[182] Sethi S., Alcid D., Kesarwala H., Tolan R.W. (2009). Probable congenital babesiosis in infant, new jersey, USA. Emerg. Infect. Dis..

[183] Joseph J.T., Purtill K., Wong S.J., Munoz J., Teal A., Madison-Antenucci S., Horowitz H.W., Aguero-Rosenfeld M.E., Moore J.M., Abramowsky C. (2012). Vertical transmission of *Babesia microti*, United States. Emerg. Infect. Dis..

[184] New D.L., Quinn J.B., Qureshi M.Z., Sigler S.J. (1997). Vertically transmitted babesiosis. J. Pediatr..

[185] Abittan B., Nizam A., Oey M., Callan F., Simmonds L., Pachtman S.L. (2019). A case of babesiosis in a pregnant patient treated with red blood cell exchange transfusion. Case Rep. Obstet. Gynecol..

[186] Feder H.M., Lawlor M., Krause P.J. (2003). Babesiosis in pregnancy. N. Engl. J. Med..

[187] Pashankar R., Prabulos A.M., Feder H.M. (2022). A woman pregnant with twins has fever, haemolysis, and thrombocytopenia caused by babesiosis: Could be confused with HELLP syndrome. Lancet.

[188] Mupombwa T., Mulla B.M., Kirby J.E., O’Brien B. (2016). *Babesia microti* Infection in Pregnancy Mimicking HELLP Syndrome. J. Bacteriol. Parasitol..

[189] Gulersen M., Brost B.C., Bobrovnikov V., Bornstein E. (2016). Acute babesiosis in pregnancy: A novel imitator of hemolysis, elevated liver enzymes, and low platelet count syndrome. Obstet. Gynecol..

[190] Luckett R., Rodriguez W., Katz D. (2014). Babesiosis in pregnancy. Obstet. Gynecol..

[191] Khangura R.K., Williams N., Cooper S., Prabulos A.M. (2019). Babesiosis in pregnancy: An imitator of HELLP syndrome. AJP Rep..

[192] Remesar S., Arnal J.L., Gómez A., Prieto A., García-Dios D., Benito A., Panadero R., Morrondo P., Díaz P. (2022). A case report of fatal feline babesiosis caused by *Babesia canis* in north western Spain. BMC Vet. Res..

[193] Sudan V., Jaiswal A.K., Srivastava A., Saxena A., Shanker D. (2015). A rare clinical presentation of transplacental transmission and subsequent abortion by *Babesia (Theileria) equi* in a mare. J. Parasit. Dis..

[194] Fukumoto S., Suzuki H., Igarashi I., Xuan X. (2005). Fatal experimental transplacental *Babesia gibsoni* infections in dogs. Int. J. Parasitol..

[195] Omar A., Grenier L.N., Marquez O., Faber S., Darling E.K. (2024). Perinatal transmission of Lyme disease: A qualitative study investigating the research priorities of patients with Lyme disease in pregnancy. PLoS ONE.

[196] Moro M.H., Bjornsson J., Marietta E.V., Hofmeister E.K., Germer J.J., Bruinsma E., David C.S., Persing D.H. (2001). Gestational attenuation of Lyme arthritis is mediated by progesterone and IL-4. J. Immunol..

[197] Carter A.M. (2020). Animal models of human pregnancy and placentation: Alternatives to the mouse. Reproduction.

[198] Challis J.R., Heap R.B., Illingworth D.V. (1971). Concentrations of oestrogen and progesterone in the plasma of non-pregnant, pregnant and lactating guinea-pigs. J. Endocrinol..

[199] Mitchell B.F., Taggart M.J. (2009). Are animal models relevant to key aspects of human parturition?. Am. J. Physiol.-Regul. Integr. Comp. Physiol..

[200] Dobbing J., Sands J. (1970). Growth and development of the brain and spinal cord of the guinea pig. Brain Res..

[201] Nacher J., Palop J.J., Ramirez C., Molowny A., Lopez-Garcia C. (2000). Early histological maturation in the hippocampus of the Guinea pig. Brain Behav. Evol..

[202] Nitsos I., Rees S. (1990). The effects of intrauterine growth retardation on the development of neuroglia in fetal guinea pigs. An immunohistochemical and an ultrastructural study. Int. J. Dev. Neurosci..

[203] Sturrock R.R. (1980). Myelination of the mouse corpus callosum. Neuropathol. Appl. Neurobiol..

[204] Pozarska A., Rodríguez-Castillo J.A., Surate Solaligue D.E., Ntokou A., Rath P., Mižíková I., Madurga A., Mayer K., Vadász I., Herold S. (2017). Stereological monitoring of mouse lung alveolarization from the early postnatal period to adulthood. Am. J. Physiol. Lung Cell Mol. Physiol..

[205] Mund S.I., Stampanoni M., Schittny J.C. (2008). Developmental alveolarization of the mouse lung. Dev. Dyn..

[206] Lechner A.J., Banchero N. (1982). Advanced pulmonary development in newborn guinea pigs (*Cavia porcellus*). Am. J. Anat..

[207] Langston C., Kida K., Reed M., Thurlbeck W.M. (1984). Human lung growth in late gestation and in the neonate. Am. Rev. Respir. Dis..

[208] Barrie S.E., Harris P. (1977). Myocardial enzyme activities in guinea pigs during development. Am. J. Physiol..

[209] Hoerter J.A., Ventura-Clapier R., Kuznetsov A. (1994). Compartmentation of creatine kinases during perinatal development of mammalian heart. Mol. Cell Biochem..

[210] Caulfield A.J., Pritt B.S. (2015). Lyme disease coinfections in the United States. Clin. Lab. Med..

